# Correction: Casamassimo, P.S. Emerging Evidence Supports Broader Definition of Chairside Behavior Guidance and Familial Compliance. *Healthcare* 2024, *12*, 1935

**DOI:** 10.3390/healthcare12212179

**Published:** 2024-11-01

**Authors:** Paul S. Casamassimo

**Affiliations:** 1 Division of Pediatric Dentistry, College of Dentistry, The Ohio State University, Columbus, OH 43214, USA; casamassimo.1@osu.edu; 2 Nationwide Children’s Hospital, Columbus, OH 43205, USA

## Missing Citation

In the original publication [1], Reference 30 was not cited. The citation has now been inserted in Section “4. What Is Next?”, “Paragraph 4”, and should read as follows:

The next step might be to engage stakeholders in an organized and directive process that identifies evidence in dentistry and supplements data with expert opinion, stakeholder input, reviews of the medical literature applicable to the topics, and investigation of electronic databases related to the topics. As has been largely the case in dentistry, evolution will likely be driven by trial and error and innovation, as randomized trials are limited and will likely require time and money. Dentistry would do well to follow medicine’s lead in addressing social determinants and system limitations to achieve health and equity. An analogous effort to this is the MORE Project [30], an integrative medical–dental model of care that presumes the benefit of physician–dentist interaction and has been implemented so that data can be derived and applied to sites other than those carefully structured in the project.

## Newly Added Reference

30. Kanan, C.; Ohrenberger, K.; Bayham, M.; Raskin, S.E.; Tranby, E.P.; Boynes, S. MORE Care: An evaluation of an interprofessional oral health quality improvement initiative. *J. Public Health Dent*. **2020**, *80*, S58–S70. https://doi.org/10.1111/jphd.12407.

With this correction, the order of some references has been adjusted accordingly.

The authors state that the scientific conclusions are unaffected. This correction was approved by the Academic Editor. The original publication has also been updated.
